# Preparation of Polycarbonate-ZnO Nanocomposite Films: Surface Investigation after UV Irradiation

**DOI:** 10.3390/molecules27144448

**Published:** 2022-07-12

**Authors:** Babak Jaleh, Sara Hamzehi, Reza Sepahvand, Saeid Azizian, Mahtab Eslamipanah, Reza Golbedaghi, Alireza Meidanchi, Rui Fausto

**Affiliations:** 1 Physics Department, Bu-Ali Sina University, Hamedan 65174, Iran; mahtabes740@gmail.com; 2 Physics Department, Lorestan University, Khorramabad, 68151 Iran; sarahamzeh1984@gmail.com (S.H.); r.sepahvand@gmail.com (R.S.); 3 Department of Physical Chemistry, Faculty of Chemistry, Bu-Ali Sina University, Hamedan 65174, Iran; sazizian@basu.ac.ir; 4 Department of Chemistry, Payame Noor University (PNU), Tehran 19395-4697, Iran; 5 Department of Physics, Payame Noor University (PNU), Tehran 19395-4697, Iran; meidanchi@pnu.ac.ir; 6 CQC-IMS, Department of Chemistry, University of Coimbra, 3004-525 Coimbra, Portugal

**Keywords:** nanocomposite, polycarbonate, ZnO nanoparticles, surface properties, UV irradiation

## Abstract

Polycarbonate (PC)-ZnO films with different percentages of ZnO were prepared by a solution stirring technique and subjected to ultraviolet (UV; *λ* = 254 nm) irradiation. Structural parameters of the samples and the effects of UV irradiation on the surface properties of the PC and PC-ZnO nanocomposites were evaluated by X-ray diffraction (XRD), X-ray photoelectron spectroscopy (XPS), atomic force microscopy (AFM), water contact angle (WCA) measurements, and a Vickers microhardness (H_V_) tester. The XRD patterns of the nanocomposite films were found to show an increase in crystallinity with the increasing ZnO nanoparticles percentage. The WCA was found to be reduced from 90° to 17° after 15 h of UV irradiation, which could be ascribed to the oxidation of the surface of the samples during the irradiation and exposure of the ZnO nanoparticles, a result that is also supported by the obtained XPS data. The microhardness value of the PC-ZnO films including 30 wt.% ZnO enhanced considerably after UV radiation, which can also be attributed to the exposition of the ZnO nanoparticles after photodegradation of the PC superficial layer of the nanocomposite films.

## 1. Introduction

Polymer composites are a class of materials with prominent physicochemical properties, where a polymer acts as the matrix and micro-, macro-, or nanomaterials as the filler [1]. Researchers have reported that nanofillers (e.g., carbon nanotubes or nano-semiconductors) can improve some features of polymers, such as their electrical conductivity and mechanical behavior [2,3,4,5,6]. Accordingly, polymer nanocomposites (PNCs) are interesting for the industry due to their enhanced properties, and have been receiving applications in fields such as environmental remediation, energy storage, and biomedicine [7]. PNCs have also been used successfully in solar cells and light-emitting diodes because of their enhanced thermal conductivity properties [8]. There are different approaches for the preparation of PNCs, such as sol–gel, mixing with inorganic filler, solution processing, extrusion, and melt processing [9].

Polycarbonate (PC) is an amorphous thermoplastic polymer with a high hardness and transparency. It is known as an “optical polymer” and has been used in a variety of optical systems due to its chemical stability and good optical properties. In recent years, the optical and mechanical features of PC-based nanocomposites have been evaluated [10,11,12,13], with different materials such as metal oxides and ceramics being used as nanofillers [13,14,15,16,17].

The changes in the surface of polymers after UV radiation have been the subject of intense investigation because UV light can damage the PNCs [18,19], strongly affecting their mechanical properties, especially plasticity. During the UV irradiation of PC, the carbonate groups absorb UV light, inducing chain-scission and free-radical formation. Consequently, small molecules can crosslink with the free-radicals, affecting the structure and properties of the polymer [20]. To prevent the degradation of polymers subjected to UV radiation, the use of UV stabilizers (UV-filters) is one of the most commonly used approaches [21]. Zinc oxide (ZnO) powder is a well-known UV-filter for polymer protection against UV radiation. ZnO nanoparticles (NPs) are a nontoxic and low-cost semiconductor with two different crystalline structures with specific physical and chemical properties: hexagonal wurtzite and cubic zinc blende; the wurtzite form is more stable and, hence, more appropriate for practical uses [22]. Generally, ZnO NPs with a 3.3 eV bandgap can absorb UV light and, thus, are widely used in sunscreen and the cosmetics industries, UV light-emitting devices, and photocatalytic processes [23].

Another important property of polymers is wettability. Controlling the wettability of surfaces is very important for some industries, such as the petroleum and brine industries [24,25]. Wettability measurement via contact angle (CA) evaluation is a common route for the study of the dynamic and static properties of the surfaces of solids [26]. Generally, CA is measured between a solid surface and a liquid (frequently water) droplet, which depends on surface characteristics such as roughness, contamination, deformation, and chemical composition. When the measured CA is higher than 90°, the solid surface is hydrophobic, while a CA smaller than 90° indicates a hydrophilic surface [27,28]. To change the wettability, various techniques have been used for the modification of surfaces such as heat treatment, chemical methods, UV–Vis irradiation, and laser/plasma treatment. According to some reports, the presence of semiconductor materials such as ZnO and TiO_2_ NPs is effective in wettability improvement [20,29,30].

Considering their useful properties, TiO_2_ NPs and ZnO NPs have been utilized as nanofillers in PNCs to enhance the mechanical and optical properties [31]. For instance, Eskandari et al. reduced the bandgap of PC by adding TiO_2_ and ZnO NPs [13]. Moreover, the hardness of the PC was increased by increasing the content of metal oxide NPs. Compared to other inorganic metal oxides, such as TiO_2_ NPs, ZnO NPs are more interesting due to their higher activity, even in small amounts [32]. There have been many reports on ZnO deposited on PC using different methods, such as the electric field-assisted chemical method [33], blade coating [34], solution mixing [35], electrochemical deposition [36,37], and radiofrequency magnetron sputtering [38]. Furthermore, the doping of the ZnO NPs with metals such as Mg, Al, Cu, and Ag has been used to enhance the physical and chemical properties of ZnO. For example, Charde and coworkers loaded Cu-doped ZnO NPs on PC (PC/ZnCu) and then evaluated the thermal, thermo-mechanical, and tribological features of the PC/ZnCu nanocomposite with interesting results [14]. In another report, Ga-doped ZnO was supported on a PC substrate by magnetron sputtering, and the chemical bonding between the Ga-doped ZnO and the PC was then being investigated by the analysis of the X-ray photoelectron spectra (XPS) of the material [39]. However, the effect of UV irradiation on the ZnO NPs composed with PC through a simple solution stirring method has not been studied previously.

Hence, in the present study, a facile physical mixing method was used for the preparation of PC-ZnO nanocomposite films with different ZnO percentages. The effects of UV light on the surface features of PC and PC-ZnO were investigated through the evaluation of surface changes (composition, roughness, wettability, hardness) and photoluminescence upon UV irradiation.

## 2. Experimental

### 2.1. Materials and Methods

PC and ZnO nanopowders (particles size < 100 nm, surface area 10–25 m^2^/g and molecular weight of 81.39) were purchased from Bayer and Aldrich, respectively. A low-pressure mercury vapor lamp (TUV 30W, Philips, Amsterdam, Holland) with an emission at 254 nm was used as the UV irradiation source at room temperature in air.

X-ray diffraction (XRD; Italstructure, ADP200) with *λ* = 1.5405 Å was used for the structure determinations. Wetting of the PC and PC-ZnO films was investigated using the CA measurement of the water droplets. The chemical information of the sample’s surface was obtained via XPS (Bestec GmbH, equipped with an Al K_α_ X-ray source at 1486.6 eV). All measurements were performed in an analyzer chamber with pressure at ~5 × 10^−8^ mbar, and binding energies were calibrated using the characteristic carbon feature C1s = 284.7 eV. Atomic force microscopy (AFM) analysis (Nanosurf Nia) was used to determine the morphology and roughness of the films. A Buehler (model 60044) microhardness tester with a microscope was used to perform the H_V_ test; the samples were indented with a Vickers diamond pyramid indenter with a square base and a 136° pyramid angle. Photoluminescence (PL) spectra were obtained in a Perkin Elmer Ls 55 luminescence spectrometer at room temperature using a xenon lamp as the excitation source (*λ* = 259 nm).

### 2.2. Synthesis of PC-ZnO Nanocomposite Films

The solvent evaporation method was used for the PC-ZnO nanocomposite film preparation. First, 1.0 g of PC was dissolved in 30.0 mL of dichloromethane using a stirrer at room temperature to obtain a uniform solution. Then, various percentages of ZnO NPs (10, 20, and 30 wt.%) were dispersed in 20 mL of dichloromethane under sonication for 30 min and added to the PC suspension and stirred for 5 h. The resulting solution was poured into a Petri dish and the PC-ZnO film was acquired through the solvent evaporation method in an oven kept at 40 °C. The obtained film had a thickness of ~100 μm.

## 3. Results and Discussion

### 3.1. XRD Characterization of ZnO NPs, and PC-ZnO Films

**Figure 1:**
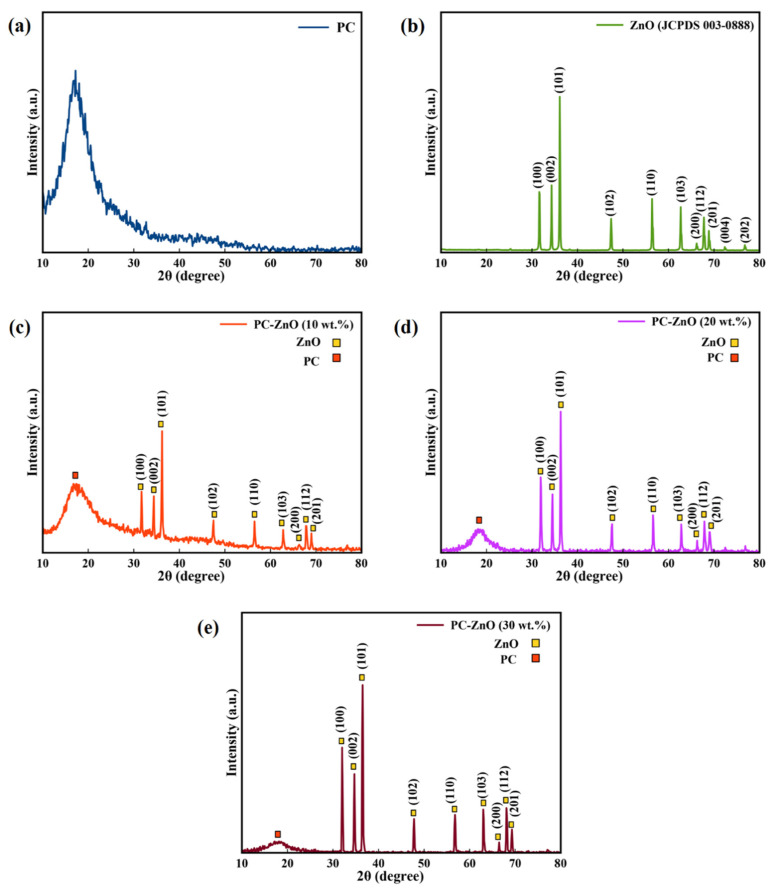
The XRD patterns of the (**a**) PC, (**b**) ZnO NPs, (**c**) PC-ZnO (10 wt.%), (**d**) PC-ZnO (20 wt.%), and (**e**) PC-ZnO (30 wt.%).

The structure of the ZnO NPs, PC, and PC-ZnO films was investigated using XRD analysis. XRD patterns of the PC and ZnO NPs samples are shown in Figure 1a,b, respectively. According to Figure 1a, the XRD pattern of PC is characterized by a broad feature extending over the 2*θ* range of 12° to 26°. For the ZnO NPs, as shown in Figure 1b, (101) was the strongest diffraction peak, indicating the dominant growth direction of the ZnO NPs. The XRD patterns obtained for the PC-ZnO films (10, 20, and 30 wt.%) are shown in Figure 1c–e.

The crystallite average diameter size (D) of the ZnO NPs in the PC-ZnO samples can be evaluated by means of Scherer’s equation [40]. A crystallite average diameter of 8.02 nm was obtained (see Table 1).

In addition, since the ZnO NPs have a hexagonal unit cell with two lattice parameters (‘a’ and ‘c’), Bragg’s law can be used to estimate the lattice parameters [41]. The calculated lattice parameters for ZnO in the PC-ZnO nanocomposite are given in Table 1, and are in agreement with those of pure ZnO (JCPDS 36-1451), indicating that the presence of PC does not influence the crystal structure of the ZnO.

### 3.2. X-ray Photoelectron Spectroscopy (XPS) Analysis

**Figure 2:**
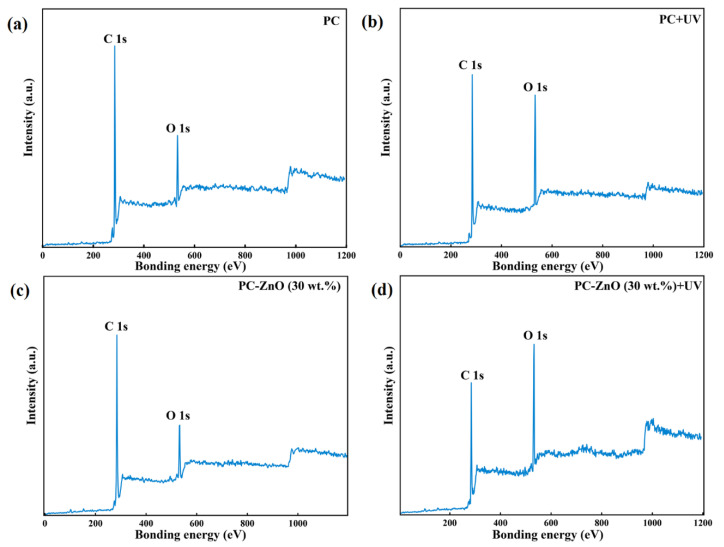
The XPS survey spectra of the (**a**) PC, (**b**) UV irradiated PC (PC + UV), (**c**) PC-ZnO (30 wt.%), and (**d**) UV irradiated PC-ZnO (30 wt.%) (PC-ZnO (30 wt.%) + UV).

XPS has been applied to evaluate the chemical state of elements and functional groups of the PC and PC-ZnO nanocomposites before and after UV treatment. The XPS spectra confirmed the presence of oxygen and carbon in the samples, as revealed by the two peaks at 284.7 eV and 532.1 eV, attributed to the C 1s and O 1s electrons, respectively. The pure PC sample had an initial amount of oxygen that increased after UV irradiation (*λ* = 254 nm, for 15 h) by ca. 19% due to oxidation of the PC film surface (Table 2 and Figure 2a,b). The XPS analysis was also performed for the PC-ZnO (30 wt.%) nanocomposite film due to its high hydrophobicity compared with the other synthesized films. As shown in Figure 2c,d, the Zn peak did not appear in the XPS spectrum of the PC-ZnO sample, suggesting that a thin layer of PC formed on the ZnO nanoparticles. Similar to the pristine PC film, the percentage of oxygen increased after UV irradiation due to the sample’s oxidation. The O percent increase was, however, larger than that observed for the PC sample, with the observed increase amounting to ca. 28% (see Table 2).

**Figure 3:**
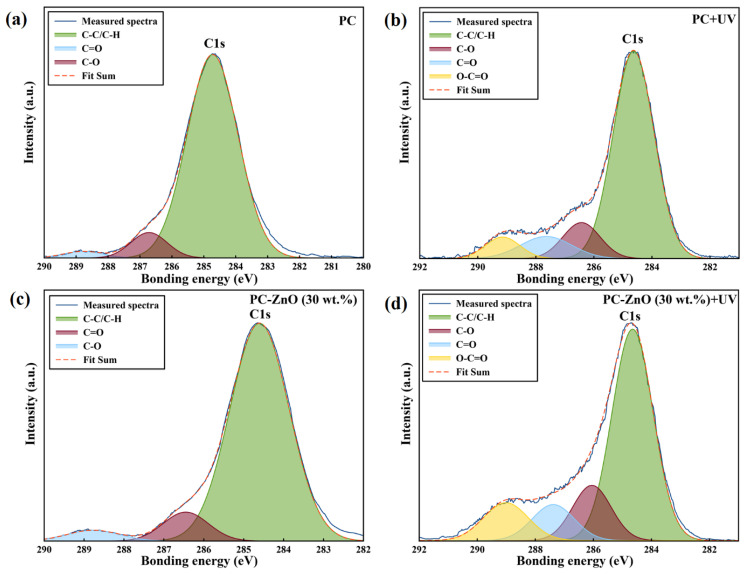
High resolution XPS spectra of the (**a**) PC, (**b**) UV irradiated PC (PC + UV), (**c**) PC-ZnO (30 wt.%), and (**d**) UV irradiated PC-ZnO (30 wt.%) (PC-ZnO (30 wt.%) + UV) in the C1s region.

**Figure 4:**
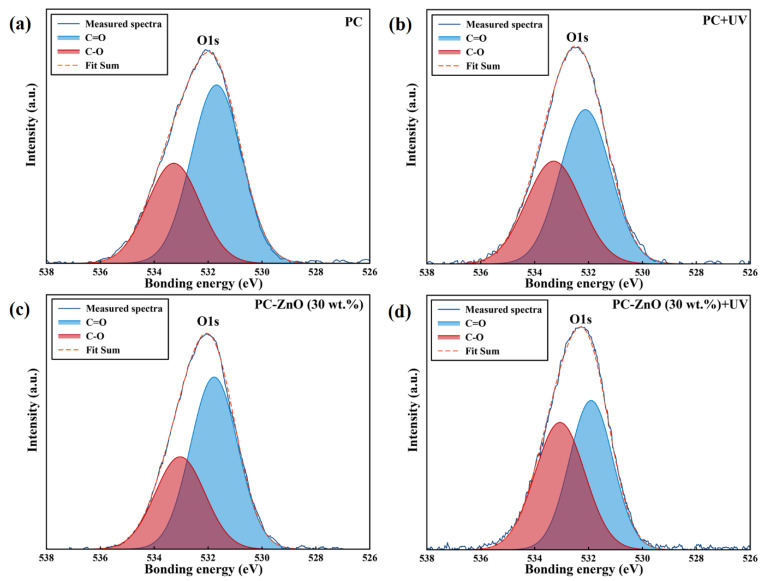
High resolution XPS spectra of the (**a**) PC, ((**b**) UV irradiated PC (PC + UV), (**c**) PC-ZnO (30 wt.%), and (**d**) UV irradiated PC-ZnO (30 wt.%) (PC-ZnO (30 wt.%) + UV) in the O1s region.

Changes in the functional groups in the samples were determined by analysis at high-resolution of the XPS spectral regions corresponding to the C1s and O1s features (Figure 3 and Figure 4). Figure 3a,c demonstrates that the C1s peak, containing three components at 284.7, 286.5 and 288.8 eV, which are assigned to carbon atoms in C–C/C–H, C–O and C=O fragments, respectively, split into four components at 284.7 eV (C–C/C–H), 286.5 eV (C–O), 287.8 eV (C=O),and 289.2 eV (O–C=O) after UV irradiation of the both PC and PC-ZnO films (Figure 3b,d). On the other hand, as seen in Figure 4, the O 1s peak of both the PC and PC-ZnO samples showed two components before and after UV irradiation at 531.7 (C=O) and 533.2 (C–O), with the component at 533.1 eV increasing in relative intensity after irradiation. The percentages of different types of bonds involving C and O, as evaluated from the relative component-band intensities of peaks of C1s and O1s, are given in Table 3 and Table 4, respectively. As seen in these tables, the UV irradiation increased the percentages of C–O and C=O bonds compared to the C–C/C–H bonds and led to the appearance of new features due to photo-oxidation (as shown in Section 3.4, these changes also cause changes in the wetting capability of the surfaces, increasing their hydrophilic character [17]).

### 3.3. AFM Measurements

**Figure 5:**
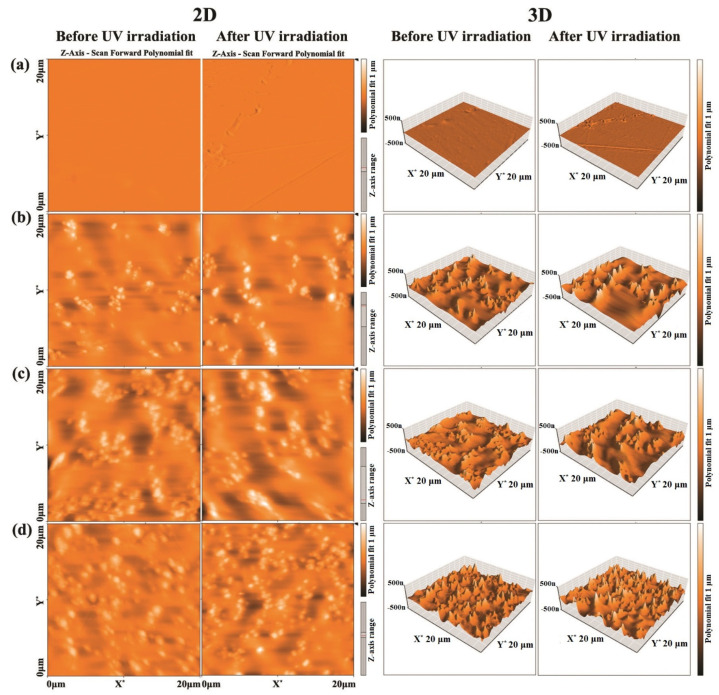
The 2D and 3D AFM images of PC-ZnO with (**a**) 0, (**b**) 10, (**c**) 20, and (**d**) 30 ZnO wt.% before and after UV irradiation.

The obtained 2D and 3D AFM images of PC-ZnO with 10, 20 and 30 ZnO wt.% before and after UV irradiation are shown in Figure 5a–d. As depicted in Figure 5a, after UV irradiation (*λ* = 254 nm, for 15 h) of the pure PC, the surface of the film became more rugged. The observed changes are characterized in more detail in Table 5, as expressed in the values of the estimated average area roughness (S_a_) and root mean square roughness (S_q_). As seen in the table, for the pristine PC sample, the area roughness increased upon UV irradiation: S_a_, from 1.7 to 2.7 nm, and S_q_, from 2.9 to 5 nm. The addition of ZnO to PC caused a considerable increase in the surface roughness (see Figure 5b–d and Table 5), which increased even more after UV irradiation for all ZnO wt.%. Therefore, it can be concluded that both the increase in the ZnO percentage and UV-irradiation substantially increased the PC surface roughness.

### 3.4. Surface Wettability

The surface wettability of the samples was measured using the water droplet method. The water contact angle (WCA) was measured for the PC and PC-ZnO 30 wt.% samples before and after different times (4 h, 8 h, 10 h, 12 h, and 15 h) of exposure to UV irradiation. The WCA of five water drops was measured in different positions of the surfaces and averaged. The results are given in Table 6.

The WCA on the non-irradiated PC-ZnO nanocomposite (90°) was found to be similar to that measured on the pristine non-irradiated PC surface (91%). However, UV irradiation was found to be more efficient in reducing the WCA in the case of the nanocomposite (from 90 to 17°, after 15 h of irradiation) than for the pure PC film (91 to 27°), which was due to the presence of ZnO NPs on the film surface after photodegradation of the thin layer of PC by the UV radiation. As concluded from the XPS results analysis made in Section 3.2, following the film photodegradation process, functional groups such as carboxylate and carboxylic acid moieties appeared on the sample surface, leading to an increase in the polarity of the surface, and, consequently, an increase in its hydrophilicity.

When ZnO NPs are placed on the PC-ZnO films, the wetting changes can be described by the following mechanisms [42]:

Electron-(e^−^)/hole-(h^+^) pairs are formed on the ZnO surface when the energy of the UV light is higher than or equal to the ZnO bandgap:

ZnO + 2hυ → 2h^+^ + 2e^−^ (1)

The surface oxygen vacancies are then produced through the reaction between some of the holes and lattice oxygen (O^2−^):

O^2−^ + h^+^ → O^1−^ (2)

O^1−^ + h^+^ → ½ O_2_ + V_O_ (oxygen vacancy) (3)

Water and oxygen compete to adsorb on these defect sites. In particular, water molecules can be coordinated into the V_O_ sites, causing the dissociative adsorption of the water molecules on the surface. These defective sites are more suitable for hydroxyl group (OH^−^) adsorption compared with oxygen adsorption [42,43]. Accordingly, the wetting of the irradiated surface including ZnO NPs is changed from a hydrophobic to a hydrophilic state due to the adsorption of OH^−^ groups. Therefore, the WCA of the PC-ZnO is drastically reduced after UV treatment.

### 3.5. Microhardness

The Vickers hardness number (H_v_) for the studied samples was determined at the load of 50 g for a constant loading time of 30 s (Table 7). An increase in the microhardness value was observed with increasing ZnO NP content in the composite. Irradiation of the pristine PC material did not significantly change the microhardness of the sample. On the other hand, for the nanocomposite materials, due to the ZnO NPs appearance at the surface due to the UV-induced photodegradation (as above-mentioned), the microhardness of the PC-ZnO samples was enhanced considerably after 15 h of UV irradiation, with the larger increase observed for the sample with the highest content of ZnO NPs (see Table 7).

### 3.6. Photoluminescence Studies

**Figure 6:**
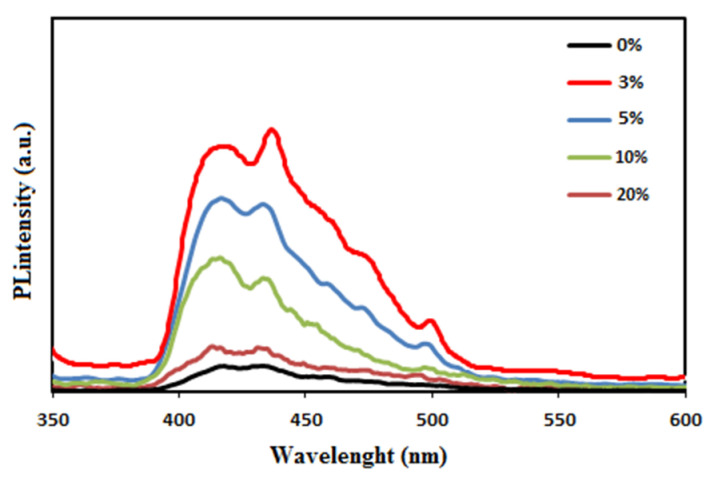
The transient PL spectra of the PC-ZnO nanocomposite films.

Figure 6 displays the transient PL spectra of the PC-ZnO films at room temperature. The main features were observed at 426, 440 and in the 476 nm region, and were seen for all the studied samples. The band at 426 nm was a typical near band edge UV emission of ZnO, indicating direct recombination of excitons through an exciton–exciton collision process [44]. The peak observed at 440 nm after the addition of ZnO corresponded to the blue-violet emission of ZnO NPs, most probably originating from zinc interstitial and zinc vacancy point defects [45,46]. The bands around 476 nm (blue) resulted from transitions involving interstitial oxygen. In addition, a green emission at 500 nm was observed, which was attributed to the transmission of an electron near the conductive band to a deeply trapped hole in the oxygen vacancy without electrons. Moreover, it was ascribed to doubly charged oxygen vacancy centers at the surface [47,48,49]. According to the obtained data, the PL intensities of the PC-ZnO (3 wt.%) were enhanced compared to PC, and then gradually decreased by increasing the ZnO NP content (10, 20 and 30 wt.%). The interactions of PC with the ZnO NPs caused the latter create an alternative pathway for the relaxation of the excited electrons, resulting in a smaller probability of radiative emission [50].

## 4. Conclusions

In summary, a simple solution mixing method was employed for the preparation of PC and PC-ZnO films, which were subsequently subjected to UV irradiation (*λ* = 254 nm). The films were characterized using XRD, XPS, AFM, and a Vickers microhardness tester.

The XRD patterns of ZnO NPs are consistent with the hexagonal phase of ZnO and show that the nanocomposite films had an increased degree of crystallinity with the ZnO nanoparticle percentage.

According to the XPS spectra, the oxygen content at the surface of the PC-ZnO films increased upon UV irradiation, which created a hydrophilic surface.

AFM analysis showed that the roughness of the PC-ZnO nanocomposites increased due to the exposure of ZnO NPs after UV irradiation.

Wetting changes of the PC and PC-ZnO nanocomposites after irradiation were also investigated by measurement of the WCA. The WCA on the non-irradiated PC-ZnO nanocomposite was found to be similar to that measured on the pristine non-irradiated PC surface. However, UV irradiation was found to be more efficient in reducing the WCA in the case of the nanocomposite (from 90 to 17°, after 15 h of irradiation) than for the pure PC film (91 to 27°). The observed super-hydrophilicity of the PC-ZnO nanocomposites after 15 h of UV irradiation was attributed to the presence of ZnO NPs on the film surface after photodegradation of the thin layer of PC by UV radiation.

## Figures and Tables

**Table 1:** The calculated crystallite size and lattice parameters of the PC-ZnO nanocomposite.

Sample	2*θ*	*hkl*	*d_hkl_* (Å)	D (nm)	Lattice Parameters		
					*c* (Å)	*a* (Å)	*c*/*a*
Pure ZnO	31.77 34.42	100 002	2.814 2.603	36	5.207	3.250	1.602
PC-ZnO (10 wt.%)	31.70	100	2.812	29	5.189	3.255	1.594
	34.53	002	2.594				
PC-ZnO (20 wt.%)	31.90 34.50	100 002	2.29 2.60	29	5.202	3.248	1.601
PC-ZnO (30 wt.%)	32.04	100	2.79	37	5.164	3.222	1.602
	34.70	002	2.58				

**Table 2:** The C and O percentages (and O/C ratio) in the PC and PC-ZnO (30 wt.%) samples before and after UV irradiation, as determined from the XPS spectra.

Sample	C	O	O/C
PC	69.50%	30.50%	0.48
PC + UV	50.69%	49.31%	0.97
PC-ZnO (30 wt.%)	71.70%	28.30%	0.40
PC-ZnO (30 wt.%) + UV	42.67%	57.33%	1.34

**Table 3:** The percentages of C bonds in the PC and PC-ZnO (30 wt.%) samples before and after UV irradiation, as determined from the high resolution XPS analysis of the C1s peak.

Sample	C–C/C–H	C–O	C=O	O–C=O
PC	90.00%	7.80%	2.20%	-
PC + UV	72.65%	9.64%	11.40%	6.31%
PC-ZnO (30 wt.%)	87.40%	3.80%	8.80%	-
PC-ZnO (30 wt.%) + UV	61.97%	10.80%	15.15%	12.08%

**Table 4:** The percentages of O bonds in the PC and PC-ZnO (30 wt.%) samples before and after UV irradiation, as determined from the high resolution XPS analysis of the C1s peak.

Sample	C=O	C–O
PC	63.26%	36.74%
PC + UV	58.00%	42.00%
PC-ZnO (30 wt.%)	63.84%	36.16%
PC-ZnO (30 wt.%) + UV	50.60%	49.39%

**Table 5:** The area roughness parameters (average area roughness, S_a_, and root mean square roughness, S_q_) of the AFM images of PC-ZnO with different ZnO wt.%.

PC-ZnO (wt.%)	Before UV Irradiation		After UV Irradiation	
	S_a_ (nm)	S_q_ (nm)	S_a_ (nm)	S_q_ (nm)
0	1.7	2.9	2.7	5.0
10	29.5	43.1	44.5	64.4
20	54.6	80.3	68.1	92.0
30	71.1	93.3	79.5	100.1

**Table 6:** The measured water contact angle (WCA) for the pristine and ZnO NPs containing (ZnO wt.30%) PC samples before and along UV irradiation.

Sample	WCA (°)
PC, PC-ZnO	91, 90
PC, PC-ZnO 4 h irradiation	58, 44
PC, PC-ZnO 8 h irradiation	41, 28
PC, PC-ZnO 10 h irradiation	32, 25
PC, PC-ZnO 12 h irradiation	31, 20
PC, PC-ZnO 15 h irradiation	27, 17

**Table 7:** The obtained microhardness values (Vickers’ parameter H_v_) for the studied samples.

Sample	Microhardness (H_V_)
PC	11.5
PC-ZnO (20 wt.%)	12.7
PC-ZnO (30 wt.%)	14.33
PC + UV	11.4
PC-ZnO (20 wt.%) + UV	13.7
PC-ZnO (30 wt.%) + UV	20.13
